# Anti-reflux mucosal ablation for refractory gastroesophageal reflux disease after Roux-en-Y gastric bypass

**DOI:** 10.1055/a-2223-0499

**Published:** 2024-01-09

**Authors:** Fares Ayoub, Kalpesh K. Patel

**Affiliations:** 1 3989 Section of Gastroenterology and Hepatology, Baylor College of Medicine, Houston, United States

Roux-en-Y gastric bypass (RYGB) is the gold standard bariatric surgical intervention in obese patients with pre-operative gastroesophageal reflux disease (GERD) 1 . Several hypotheses have been proposed to explain GERD recurrence postoperatively, including retained acid-secreting parietal cells in the pouch 2 , dysmotility of the Roux limb, and herniation of the gastric pouch through the hiatus. We report the successful treatment of refractory GERD following RYGB using anti-reflux mucosal ablation (ARMA) ( Fig. 1 ).

**Fig. 1:**
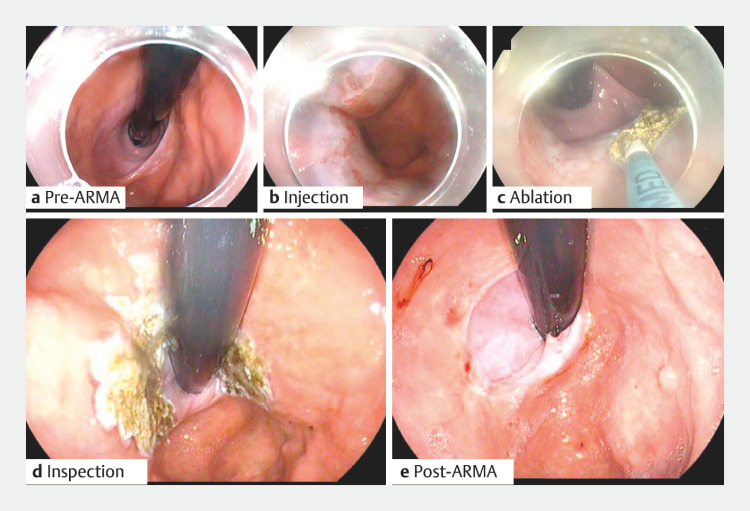
Procedural steps and outcomes. **a** Pre-intervention endoscopic view of the gastroesophageal junction. **b** Injection of submucosal lifting solution. **c** Ablation of a 180° area around the gastroesophageal junction. **d** Inspection following ablation. **e** Follow-up 4 weeks after anti-reflux mucosal ablation, with significant improvement.

A 54-year-old woman with a history of RYGB presented with symptoms of GERD, including significant regurgitation and a sour taste in her mouth, which affected her quality of life (GERD-Q score 15). Esophagogastroduodenoscopy revealed a 3-cm hiatal hernia with an American Foregut Society (AFS) hiatus classification of 4 3 , a medium-sized pouch, and a patent gastrojejunostomy. Examination of the gastroesophageal junction revealed loss of an effective flap valve. Wireless capsule pH monitoring revealed a total of 0% acid exposure time, as expected following RYGB. A barium esophagram confirmed a small sliding hiatal hernia with regurgitation. She declined surgical hiatal hernia repair for fear of adverse events. She was offered ARMA for symptom control, to which she agreed ( Video 1 ).

Anti-reflux mucosal ablation after Roux-en-Y gastric bypass.Video 1

Follow-up endoscopy 4 weeks after ARMA showed significant improvement in the hiatal defect, with an improvement in AFS classification to hiatus grade 1. At 6 months post-ARMA, she reported significant improvement in quality of life (GERD-Q score of 6), with resolution of regurgitation and no dysphagia.

Given the altered anatomy after RYGB, management of refractory GERD can be challenging. Laparoscopic hiatal hernia repair and radiofrequency ablation of the gastroesophageal junction (Stretta procedure) have been reported 4 . Our case demonstrates that ARMA may be an additional therapeutic option. First described by Hernández Mondragón et al. 5 in 2020, ARMA likely exhibits its beneficial effects by remodeling the gastroesophageal junction, leading to decreased proximal gastric distensibility.

Endoscopy_UCTN_Code_TTT_1AO_2AJ

## Notes

Endoscopy E-Videos https://eref.thieme.de/e-videos
*E-Videos* is an open access online section of the journal *Endoscopy* , reporting on interesting cases and new techniques in gastroenterological endoscopy. All papers include a high-quality video and are published with a Creative Commons CC-BY license. Endoscopy E-Videos qualify for HINARI discounts and waivers and eligibility is automatically checked during the submission process. We grant 100% waivers to articles whose corresponding authors are based in Group A countries and 50% waivers to those who are based in Group B countries as classified by Research4Life (see: https://www.research4life.org/access/eligibility/ ). This section has its own submission website at https://mc.manuscriptcentral.com/e-videos .
